# Transient lymphocyte count decrease correlates with oncolytic adenovirus efficacy in humans: mechanistic and biomarker findings from TUNIMO phase I trial

**DOI:** 10.1136/jitc-2024-010493

**Published:** 2025-01-27

**Authors:** Santeri A Pakola, James H A Clubb, Tatiana V Kudling, Mirte van der Heijden, Elise Jirovec, Victor Arias, Lyna Haybout, Katriina Peltola, Tuomo Alanko, Jorma Sormunen, Teijo Pellinen, Kristian Taipale, Dafne C A Quixabeira, Claudia Kistler, Riikka Havunen, Suvi Sorsa, Joao M Santos, Victor Cervera-Carrascon, Akseli Hemminki

**Affiliations:** 1 Cancer Gene Therapy Group, Translational Immunology Research Program, University of Helsinki, Helsinki, Finland; 2 TILT Biotherapeutics Ltd, Helsinki, Finland; 3 Comprehensive Cancer Center, HUS Helsinki University Hospital, Helsinki, Finland; 4 Docrates Cancer Center, Helsinki, Finland; 5 Digital Microscopy and Molecular Pathology Unit, University of Helsinki Institute for Molecular Medicine, Helsinki, Finland; 6 Health and Hospital Services, Wellbeing Services County of North Karelia – Siun sote, Joensuu, Finland

**Keywords:** Oncolytic virus, Biomarker, Solid tumor, Gene therapy

## Abstract

**Background:**

Oncolytic viruses (OVs) are promising immunotherapeutics to treat immunologically cold tumors. However, research on the mechanism of action of OVs in humans and clinically relevant biomarkers is still sparse. To induce strong T-cell responses against solid tumors, TILT-123 (Ad5/3-E2F-d24-hTNFa-IRES-hIL2, igrelimogene litadenorepvec) was developed. TILT-123 encodes two transgenes: tumor necrosis alpha (TNFa) and interleukin-2 (IL-2). TUNIMO (NCT04695327) was a phase I clinical trial using TILT-123 in patients with advanced solid tumors aiming to assess the safety, efficacy, and immunological effects of TILT-123. Research presented in this study evaluated the immunological effects of TILT-123 in the TUNIMO trial by using biological samples collected from the patients during the study, with an objective to leverage the findings to develop possible biomarkers of response and gain insights into possible synergistic combination treatments.

**Methods:**

20 patients with advanced solid tumors were treated with TILT-123. Response to therapy was assessed with contrast-enhanced CT and fluorodeoxyglucose positron emission tomography, along with overall survival (OS) calculation. Biological samples from patients were collected in the form of blood and tumor biopsies. Collected samples were analyzed with immunohistochemistry, transcriptomics, proteomics, and flow cytometry.

**Results:**

TILT-123 induced cyclical decreases in blood lymphocyte count, and more substantial blood lymphocyte count correlated with better radiographical response and longer OS. Lymphocyte count findings were confirmed with external control dataset of 96 patients. More substantial lymphocyte count change was linked to stronger immune activation in plasma proteome after intravenous TILT-123 and the presence of TILT-123 mRNA in tumors. Regarding other assays. tumor biopsies profiled showed increased amounts of CD8+ T cells, CD4+ T cells and NK cells after intravenous TILT-123, but not after intratumoral TILT-123. Transcriptional differences were seen in tumors after intravenous therapy and intratumoral therapy, with patients benefitting therapy showing stronger downregulation of immune activation at all time points.

**Conclusions:**

TILT-123 therapy induced accumulation of effector lymphocytes in tumors. Peripheral lymphocyte count decrease is a promising biomarker for assessing oncolytic adenovirus therapy response.

**WHAT IS ALREADY KNOWN ON THIS TOPIC**

- Oncolytic viruses are a promising anticancer therapeutic, and multiple clinical trials are underway in different cancer indications. However, immunological evaluation of the mechanism of action of oncolytic viruses in humans, and identification of relevant biomarkers are poorly understood aspects. Our study aimed to characterize the immunological effects of TILT-123, an oncolytic adenovirus encoding for tumor necrosis alpha and interleukin-2, in a phase I trial of advanced solid tumors, and establishing clinically relevant biomarkers of response.

**WHAT THIS STUDY ADDS**

- Transient lymphocyte decrease has been known to be a side effect of adenoviral therapeutics and a phenomenon associated with virus infections in general. We are the first to show that acute lymphocyte decrease after oncolytic adenovirus therapy correlates with therapeutic efficacy.

**HOW THIS STUDY MIGHT AFFECT RESEARCH, PRACTICE OR POLICY**

- Our study highlights that efficacy of oncolytic adenovirus treatment is linked to lymphocyte count decrease in the peripheral blood. Lymphocyte counting is available in most hospitals, providing an easy and cost-effective way of monitor oncolytic adenovirus efficacy. The other assays conducted provide rationale for combination treatments with oncolytic adenovirus therapy—such as NK cell or T cell-based therapies, where trafficking of lymphocytes to tumors could be beneficial for treatment efficacy.

## Introduction

 Immunotherapy in the form of immune checkpoint inhibitors (ICIs) and chimeric antigen T cells (CAR T cells) revolutionized cancer care in the 2010s and is now considered a foundational form of cancer therapy alongside surgery, traditional chemotherapy, hormonal, targeted and radiation therapies. However, ICIs and adoptive cell therapies have not been able to mount responses in all types of cancers, nor does every patient benefit in a given tumor type. Oncolytic viruses (OVs) have been suggested as a suitable option to combat immunotherapy resistance in solid cancers, due to their ability to elicit strong inflammatory responses and versatility in delivering transgenes directly into tumors.^1^ However, at this time, it is not clear which patients optimally benefit from OVs and few usable clinical biomarkers have been identified.^2^

TILT-123 (Ad5/3-E2F-d24-hTNFa-IRES-hIL2, igrelimogene litadenorepvec) is an OV developed with the aim to induce T-cell infiltration into tumors and support their cytotoxicity and proliferation within tumors. The transgenes of TILT-123, tumor necrosis factor-alpha (TNFa) and interleukin-2 (IL-2), were chosen after preclinical evaluation of different immunostimulatory cytokines against the backdrop of learnings from an individualized treatment program where 290 patients were treated with 10 different oncolytic adenoviruses.^3 4^ TUNIMO (NCT04695327) was a phase I clinical trial of TILT-123 monotherapy in advanced solid tumors, aiming to study the safety, efficacy and immunological effects of TILT-123. Patients received a single intravenous dose of TILT-123, followed by five intratumoral doses. Treatments were well tolerated, and disease control was seen in 60% of patients evaluable with positron emission tomography (PET) criteria. Long overall survival was seen in selected patients.^5^

Collection and analysis of patient specimens from clinical trials is crucial for early-phase therapeutics since these offer unique insights into the therapy which might not be unraveled in animal or ex vivo models.^6^ Additionally, as more OVs therapeutics are reaching late-stage clinical trials and possible clinical approval, there is a growing need for tools which aid in the selection of patients likely to benefit.^7^ Immunotherapies, and especially highly proinflammatory OVs such as TILT-123, are prone to pseudo-progression when assessed with size based approaches such as computer tomography, and thus better tools would be needed for physicians to distinguish pseudoprogression from true progression.^8 9^

Currently, OV therapy has few human biomarkers predicting therapy success.^7^ Preclinical research has identified markers of improved cancer cell infection by OVs, where interferon signature, metabolic pathways and surface expression of entry receptors have shown correlations to infectivity of OVs.^10 11^ These markers are however of limited use in the clinic, where direct samples from tumors are challenging to obtain, and samples may not represent the totality of tumor, especially when considering time-dependent tumor evolution due to environmental pressures.^12 13^

A recent landmark study in patients with recurrent glioblastoma multiforme treated with oncolytic HSV-1 (CAN-3110, rQNestin34.5v.2) was able to link multiple intratumoral and peripheral antiviral immune changes to longer patient survival.^14^ In fact, the field of OVs has studied antiviral immune responses for multiple decades, most commonly in the form of a neutralizing antibody assay, where the ability of patient’s serum to block vector transduction in vitro is measured.^7 15^ However, the justification behind the neutralization assays has primarily been to study the neutralization of the viral vector, and not monitor therapeutic efficacy through the assay outcome. Some studies have reported association of neutralizing antibody titer to therapeutic efficacy, and in general, the presence or induction of neutralizing antibodies has not been detrimental to therapeutic efficacy, and in many scenarios, more neutralizing antibodies have correlated with better response.^14 16 17^ However, the neutralizing antibody assay suffers from non-standardized assay conditions, in addition to inherently measuring B-cell response, and although linked to activation in other parts of the immune system, efficacy from OV treatment is thought to arise from activation of cytotoxic compartments of the immune system, more specifically cells of T and NK cell origin.^18^ Furthermore, B-cell responses take time to develop, classically understood to take weeks to reach the peak titer.^19 20^ Thus, the field of OV therapy and cancer therapy in general would benefit from biomarkers that are (1) easy to collect, preferentially from the peripheral blood, (2) correlate well to radiologic and/or survival response and (3) are standardized or easily standardizable.

Our research presented here aimed to assess the correlates of response in patients treated with TILT-123 in the TUNIMO trial. The immunostimulatory effects of TILT-123 were assessed in multiple sample types collected in the trial and these were correlated to radiological response and to overall survival outcomes.

## Methods

### Patients and clinical trial protocol

TUNIMO (NCT04695327) was a multicenter, single-arm, dose-escalation phase I trial aimed to assess the safety, efficacy, and immunogenicity of TILT-123. The trial enrolled 20 patients with advanced solid tumors. Key inclusion criteria for the trial included advanced solid tumor that had failed conventional therapy, adequate hematological (hemoglobin <100 g/L, platelets >75x10^9^/L, white cell count (WCC) >3.0×10^9^/L), hepatological (alanine transaminase and aspartate transferase <3 times the upper limit of normal and bilirubin <1.5 times the upper limit of normal) and renal status (estimated glomerular filtration rate >60 mL/min), no concurrent cancer or immunosuppressive therapy and no previous use of OVs. At least one tumor had to be available for intratumoral dosing, and patients had to have a WHO/ECOG performance status of 0 or 1 and a life expectancy longer than 3 months at screening.

Patients received a single dose of intravenous TILT-123 (dose escalation range from 3×10^9^ to 4×10^12^ viral particles (VPs)) and up to five doses of intratumoral TILT-123 (dose escalation range from 3×10^9^ to 5×10^11^ VPs). Patients were assessed with imaging at baseline and at day 78 with contrast-enhanced CT and fluorodeoxyglucose PET, using RECIST 1.1, iRECIST and PET-based response criteria. Tumor CT changes presented in this research relate to RECIST 1.1-based percentage change in the sum of the longest diameters of the target lesions. PET-based response criteria used in the trial are presented in online supplemental table 1. Biopsies for immunological assessment of tumors were collected at baseline, before the first intratumoral dose on day 8, and before the third intratumoral dose on day 36, if clinically safe as judged by the treating physician. PBMCs were extracted from pretreatment blood draws at baseline and on days 8, 36 and 64 using BD Vacutainer CPT tubes (362753, Becton, Dickinson and Company, New Jersey, USA) and cryopreserved in 10% DMSO supplemented FBS in −140°C until analysis. Serum for proteomic analysis and neutralizing antibody assessment was collected pretreatment and 16 hours post-treatment on days 1, 8, 22, 36, 50, and 64. Standard laboratory blood tests for complete blood count, liver and kidney function tests and standard electrolytes were collected pretreatment and 16 hours post-treatment at screening and on days 1, 8, 22, 36, 50, and 64 and analyzed with standard hospital laboratory equipment.

### Immunohistochemistry of biopsies

After collection, biopsies were fixed in formalin and dehydrated in 70% ethanol. Fixed biopsies were embedded in paraffin and sectioned for H&E and antibody staining. Sections were stained with three multiplexed immunofluoresences (mIF) panels of antibodies targeting CD8, CD56, PD-1, Granzyme-B, CD45, CD4, CD20, FOXP3, CD68, CD11c, PD-L1, and CD16. All three antibody panels included a pan-epithelial cocktail consisting of pan-cytokeratin targeting antibody and E-cadherin targeting antibody. All antibody panels were stained with sequential staining method described in more detail previously.^21^ Details of antibody conjugations, clones and vendors used in immunohistochemistry (IHC) are shown in online supplemental table 2. Antibody-stained sections were imaged and scanned with Zeiss Axioscan Z1 using a 20× objective (Carl Zeiss AG, Germany) and numerical values of cells were acquired with CellProfiler V.4.2.5.^22^ For analysis, if a biopsy contained less 1000 cells, the biopsy was deemed to have missed the tumor and the sample was excluded from downstream analysis. If two biopsies were available from the same patient from the same time point and injected/non-injected status, the average cell count was taken and used in analyses where overall survival was used.

### Flow cytometry of peripheral blood mononuclear cells

Cryopreserved PBMC samples were rapidly thawed in 37°C water bath, washed with 10% FBS RPMI, seeded at 7×10^5^ cells per well, rested for 1 hour in 37°C incubator, then Fc-blocked with BD Pharmingen Human BD Fc Block (564219, Becton, Dickinson and Company) for 15 min in 4°C and stained with antibodies targeting CD3, CD4, CD8, CD45RA and CD197 (CCR7) for 45 min in 4°C. Details to conjugations, clones, vendors and dilutions used in flow cytometry are shown in online supplemental table 3. Dead cell discrimination was performed using 7-AAD (420404, Biolegend, California, USA). Fluorochrome compensation was accomplished with UltraComp eBeads Compensation Beads (01-2222-42, Thermo Fisher, Massachusetts, USA) and fluorescence-minus-one and unstained controls were used for gating. Stained samples were acquired directly after staining and washing with Novocyte Quanteon (Agilent Technologies, Califonia, USA) and generated fcs-files were analyzed with FlowJo (Becton, Dickinson and Company).

### Proteomic analysis of serum

Serum collected from pretreatment and post-treatment samples was analyzed with Olink Target 96 Immuno-Oncology panel (Thermo Fisher). Relative protein levels (NPX) were normalized across runs with bridging samples. Data from proteomic analysis were analyzed with RStudio V.2023.12.0 (Posit PBC, Massachusetts, USA) using OlinkAnalyze package (V.3.8.2). Protein levels at different time points were compared with Mann-Whitney U tests.

### Transcriptomic analysis of biopsies

RNA was extracted from biopsies using RNA purification Mini Kit (740955, Macherey-Nagel, Germany). RNA samples which did not pass quality check (e.g., the concentration was below the threshold or of poor quality) were excluded from the evaluation. Extracted RNA was analyzed with NanoString nCounter PanCancer Immune Profiling Panel (NanoString Technologies, Washington, USA) with additional custom probes targeting the Ad5 hexon, Ad3 fiber and 24 bp deleted Ad5 E1A. Design of custom probes is shown in online supplemental table 4. RNA expression levels across batches were normalized with bridging samples. Analysis of transcripts detected, including gene-set analysis using Gene Ontology (GO), was done with RStudio using package clusterProfiler (V.4.10.1).^23^ Transcript counts were compared with unpaired test when comparing responders to non-responders, and with paired tests when comparing chances across time. Sample normality was tested with Shapiro test, and t-test was used for normally distributed samples and Wilcoxon signed-rank test was used for non-normally distributed samples. For survival analysis of tumor transcriptomics, a patient has deemed a long survivor if the patient was alive more than 120 days post enrolment.

### Validation patient population

Patients treated in the Advanced Therapy Access Program (ATAP) between 2007 and 2012, with available lymphocyte count data were included as an external validation dataset. A total of 96 patients were available with pretreatment and 1-day post-treatment lymphocyte counts. Patient demographics of included ATAP patients are shown in online supplemental table 5.

### Graphical illustrations and statistics

Graphs and statistics were produced with GraphPad Prism V.9.4.1. (GraphPad Software, Massachusetts, USA), RStudio V.4.3.3 (Posit PBC) and Biorender.

## Results

### Administration of TILT-123 leads to transient lymphocyte decrease in blood correlating with better radiographical response

**Figure 1:**
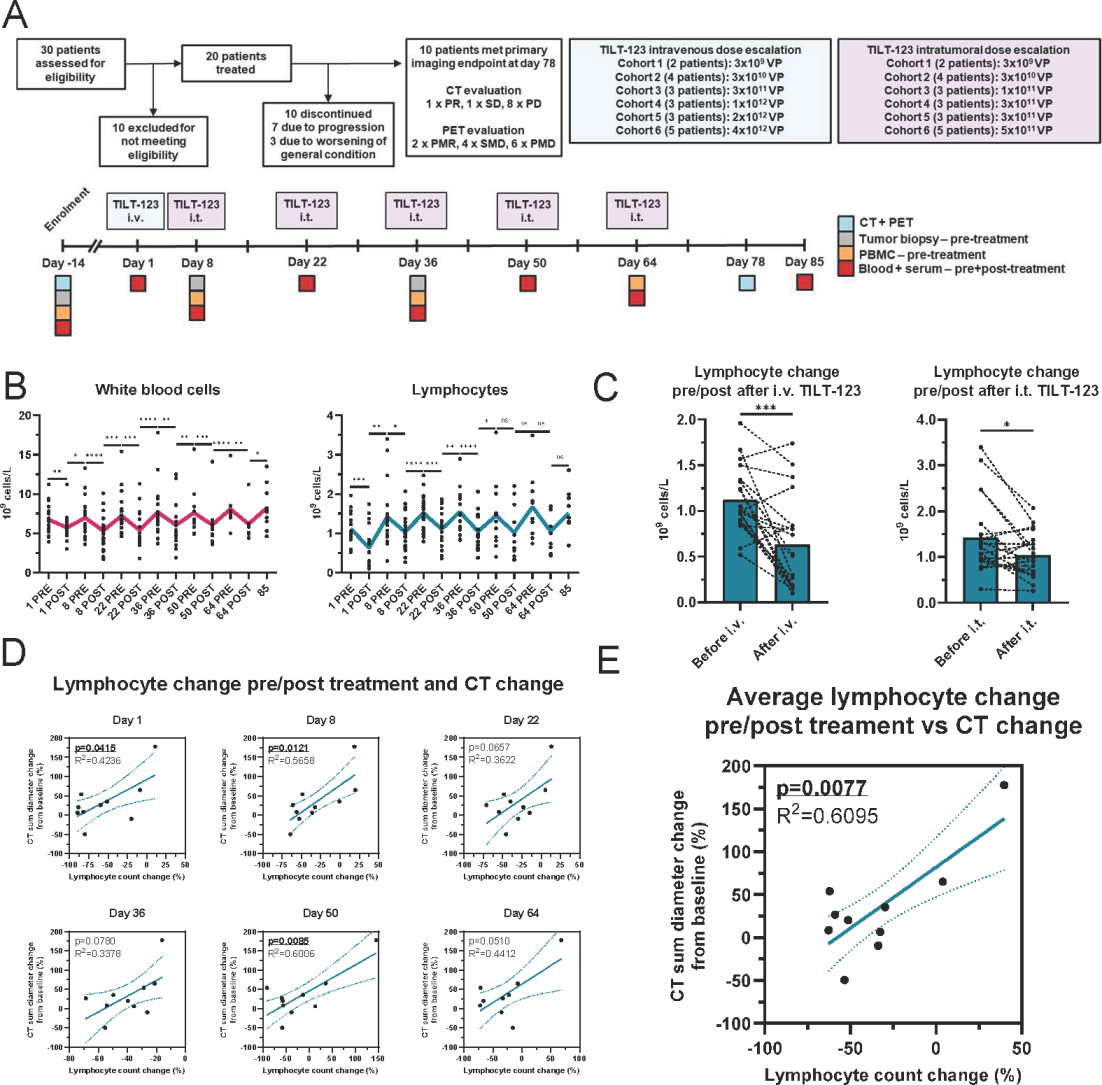
(A) TUNIMO CONSORT diagram, dose-escalation scheme, trial timeline and samples collected. (**B**) Changes in white blood cells and lymphocytes across trial. All individual datapoints shown, means presented with colored line (N=20 patients). (**C**) Change in lymphocyte count post intravenous and intratumoral TILT-123 administration (day 1 and day 8, N=20 patients on each day). (**D**) Correlation of lymphocyte count change in percentage on each treatment day to CT tumor diameter change. N=10 patients. (**E**) Correlation of average lymphocyte count change in percentage on all treatment days to CT tumor diameter change. N=10 patients. For (B, C) time points compared with Fisher’s paired t-test. For (D, E) R^2^ for goodness of fit and p value for slope deviation from zero shown. *p<0.05, **p<0.01, ***p<0.001, ****p<0.0001. CONSORT, Consolidated Standards of Reporting Trials; PD, progressive disease; PET, positron emission tomography; PMR, partial metabolic response; PMD, progressive metabolic disease; PR, partial response; SD, stable disease; SMD, stable metabolic disease.

The dose escalation phase of TUNIMO included 20 patients with advanced solid tumors refractory to standard therapy, of which 10 completed the whole planned trial including the final imaging time point. Patient characteristics, baseline clinical information, response evaluation and biopsy availability are shown in table 1. The trial Consolidated Standards of Reporting Trials diagram, dose escalation scheme, treatment scheme, collected samples and responses are shown in figure 1A. In total, patients received one intravenous administration of TILT-123, followed with five intratumoral administrations every 2 weeks from day 8 onwards. Of the 10 patients who completed the trial until the final imaging time point, one patient (20103) showed a partial response and one patient (20108) showed stable disease as per RECIST 1.1. Patients were also evaluated with PET criteria (online supplemental table 1), by which two patients showed partial metabolic responses (20103 and 20202) and four patients showed stable metabolic disease (20204, 20206, 20104 and 20108). Patients’ sum percentage changes by CT and PET are shown in table 1. Administration of TILT-123 led to transient decreases in both the WCC and the lymphocyte count (figure 1B,C). No similarly large changes could be seen for other blood cells, although small decreases were seen for platelets and monocytes (online supplemental figure 1A).

**Table 1:** Patient characteristics and responses

Patient	Cohort	ECOG	Tumor type	Stage	No. of prior systemic treatments	RECIST 1.1	% SLD change on day 78 (or extension)	PET criteria	% sum SUVmax change on day 78 (or extension)	Overall survival, days	Biopsy IHC availability (baseline/day 8/day 36)
20202	1	1	NSCLC	IV	5	PD	178	PMR	−45	102	+/+/+
20203	1	1	Melanoma of unknown primary	IV	7	–	–	–	–	66	+/+/+
20204	2	0	Myxoid liposarcoma	IV	5	PD	35.6	SMD	20	821	−/+/−
20205	2	1	Ovarian serous cystadenocarcinoma	IV	14	–	–	–	–	70	+/+/−
20101	2	1	Breast carcinoma	IV	7	–	–	–	–	56	+/−/−
20206	2	1	HG serous ovarian carcinoma	IIIC	16	PD	−9.3	SMD	−2.7	192	+/+/+
20103	3	0	Anaplastic thyroid carcinoma	IV	1	PR	−49.3 (–77.6)	PMR	−12.4 (−81.6)	983*	−/−/−
20102	3	1	Rhabdomyosarcoma	IV	3	–	–	–	–	118	−/−/−
20211	3	1	Cutaneous melanoma	IV	3	PD	65	PMD	−5.6	377	+/+/+
20212	4	1	Leiomyosarcoma	IV	4	PD	8.6	PMD	27.9	557	−/+/+
20104	4	1	Leiomyosarcoma	IV	6	PD	26.6	SMD	−1.5	206	+/+/−
20107	4	0	Chondrosarcoma	IV	2	–	–	–	–	104	−/−/−
20213	5	1	Neuroendocrine carcinoma of the bladder	IV	3	–	–	–	–	94	+/+/+
20108	5	1	Adenoid cystic carcinoma	IV	0	SD	6.7	SMD	−9.6	221	−/−/−
20214	5	1	Mucinous carcinoma of the appendix	IV	6	–	–	–	–	109	+/+/+
20216	6	1	Cutaneous melanoma	IV	2	–	–	–	–	104	+/+/+
20109	6	0	Leiomyosarcoma	IV	5	–	–	–	–	109	−/−/−
20217	6	1	Myxoid liposarcoma	IV	4	PD	54.1	PMD	120.8	131	+/−/−
20219	6	1	HG mucoepidermoid carcinoma of the parotid gland	IV	2	PD	20.3	PMD	322.9	239	+/+/+
20111	6	0	HG serous carcinoma of the peritoneum	IV	15	–	–	–	–	196	−/−/−

* Patient alive as of the data cut-off on July 27, 2024.

+biopsy collected and suitable for IHC staining−biopsy not collected or not suitable for IHC stainingIHCimmunohistochemistryNSCLCnon-small cell lung cancerPDprogressive diseasePMDprogressive metabolic diseasePMRpartial metabolic responsePRpartial responseSDstable diseaseSLDsum lesion diameterSMDstable metabolic diseaseSUVstandardized uptake value

Because TILT-123 induced transient lymphocyte and WCC decrease, we next aimed to assess if the magnitude of lymphocyte or WCC decrease on each treatment day (1, 8, 22, 36, 50 and 64) was correlated with tumor size decrease. Indeed, a larger decrease in the total lymphocyte count was associated with larger tumor size decrease on each treatment day (figure 1D). A waterfall plot of all lesions stratified by average lymphocyte count decrease is shown in online supplemental figure 1B. No correlation of WCC decrease to tumor size decrease could be seen (online supplemental figure 1C). When averaging the treatment days, the average lymphocyte count change also correlated with tumor size decrease (p=0.0077, figure 1D), and no similar correlation could be seen with the average WCC change (p=0.8898, online supplemental figure 1D). Interestingly, a larger lymphocyte count decrease on day 64 correlated also with larger SUVmax increase on imaging time point at day 78 (p=0.0498, online supplemental figure 1E), possibly suggesting an influx of glucose-consuming lymphocytes into tumors from day 64 intratumoral dosing, leading to falsely elevated SUVmax readings on day 78. While PET has been proposed as a more sensitive method for imaging therapeutic response to immunotherapies compared with CT, glucose uptake by activated lymphocytes has been a concerning caveat.^24 25^ No correlation was seen between the total WCC change on day 64 and SUVmax evaluation (p=0.9847, online supplemental figure 1E).

### TILT-123 administration leads to enhanced lymphocyte infiltration to tumors while baseline stromal levels of PD-L1 correlate with longer overall survival

**Figure 2:**
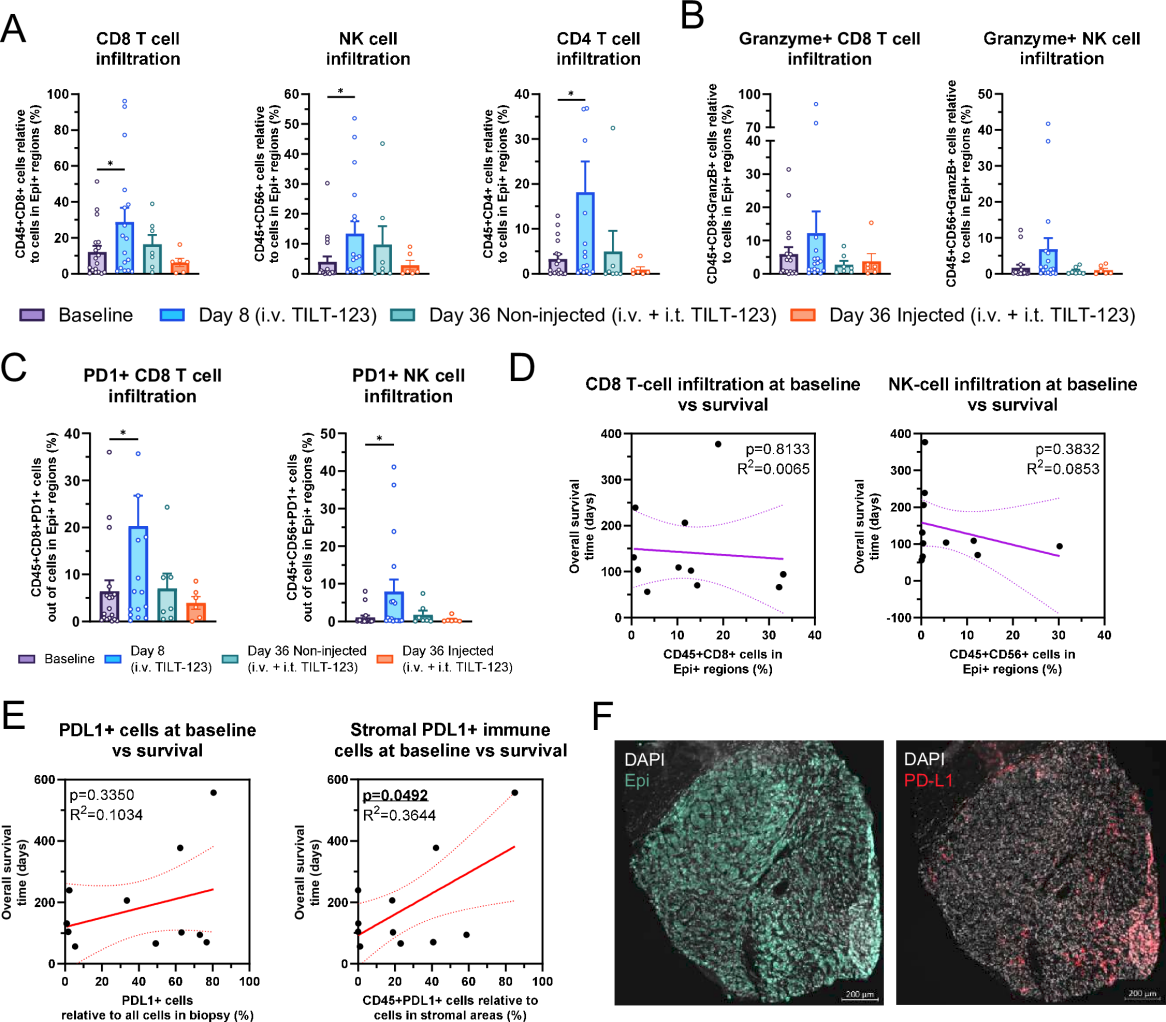
(A) Changes in intraepithelial CD8+T cells, NK cells and CD4+T cells across trial compared with Epi+DAPI+ cells in epithelial areas. (**B**) Changes in intraepithelial Granzyme B+ CD8+ T cells and NK cells across trial compared with Epi+DAPI+ cells in epithelial areas. (**C**) Changes in intraepithelial PD1+ CD8+ T cells and NK cells across trial compared with Epi+DAPI+ cells in epithelial areas. (**D**) Comparison of baseline intraepithelial CD8+T cell and NK cell amounts out of Epi+DAPI+ cells to overall survival. (**E**) Comparison of baseline PDL1 expression and stromal immune cell PDL1 expression to overall survival. (**F**) Example staining for Epi and PD-L1 in tumor biopsies in a baseline sample of patient 20104 with leiomyosarcoma. For (A–C) mean and SEM shown, groups compared with unpaired t-test. For (D, E) R^2^ for goodness of fit and p value for slope deviation from zero shown. *p<0.05.

We next evaluated immune cell changes at the tumor level. TILT-123 administration led significant increase in CD8+T cells, NK cells and CD4 T cells (p=0.0296, p=0.0367 and p=0.0212, respectively, figure 2A, N=11 patients, 1 or 2 biopsies per time point per patient) on day 8 samples, representing the effects of intravenous TILT-123. No significant changes could be seen in day 36 samples, but interestingly non-injected lesions showed higher immune cell infiltration than injected lesions.

Regarding immune cell activation status after intravenous TILT-123, a non-significant increase in Granzyme B expressing CD8+T cells and NK cells could be seen (p=0.2835 and p=0.0612, respectively, figure 2B). Of note, a significant increase in PD-1+CD8+ T cells and NK cells could be seen on day 8 (p=0.0228 and p=0.0167, respectively, figure 2C). Regarding tumor infiltration and relation to cyclical peripheral lymphocyte count decrease observed, lower baseline levels of CD8+T cells associated with larger peripheral lymphocyte count decrease after intravenous TILT-123 (p=0.0051 (online supplemental figure 2A). Regarding day 8 tumor infiltration, a larger decrease in peripheral lymphocyte count on day 1 correlated with less NK cells and CD8+T cells in filtrating into tumors (p=0.0298 and p=0.0077, online supplemental figure 2B), but interestingly smaller rebound of the lymphocyte count from day 1 post-treatment to day 8 pretreatment levels correlated with more CD8+T cells infiltrating the tumors (p=0.0172, online supplemental figure 2C). Altogether, these findings suggest that acute lymphocyte count decrease after therapy is not reflecting tumor infiltration, and that the infiltration into tumors possibly occurs later after treatment administration.

Due to limited amount of biopsy samples from patients who were imaged at day 78, we correlated baseline IHC findings to overall survival of the patients in order to assess treatment success. Baseline infiltration of CD8+T cells or NK cells did not correlate with overall survival (p=0.8133 and p=0.3832, figure 2D), suggesting that baseline tumor infiltration by lymphocytes is not relevant for TILT-123 therapy (as opposed to what has been proposed for ICIs). Additionally, baseline staining for PD-L1, a marker commonly used to identify patients for PD-1/PD-L1 checkpoint inhibitor therapy, did not predict overall survival when assessing all PD-L1 positive in the total biopsy area (figure 2E, p=0.3350). However, when assessing more compartmentalized PD-L1 expression, relative stromal expression of PDL1 on immune cells correlated with longer overall survival (figure 2E, p=0.0492). More specifically, PDL1+expression seemed to localize to CD68+CD11 c immune cells, likely representing monocyte/macrophage lineage cells (online supplemental figure 2D, p=0.0417). Exemplary staining of PD-L1 in patient 20104 is shown in figure 2F.

### TILT-123 induces different transcriptional changes in tumor biopsies after intravenous dosing, and in injected and non-injected tumors

**Figure 3:**
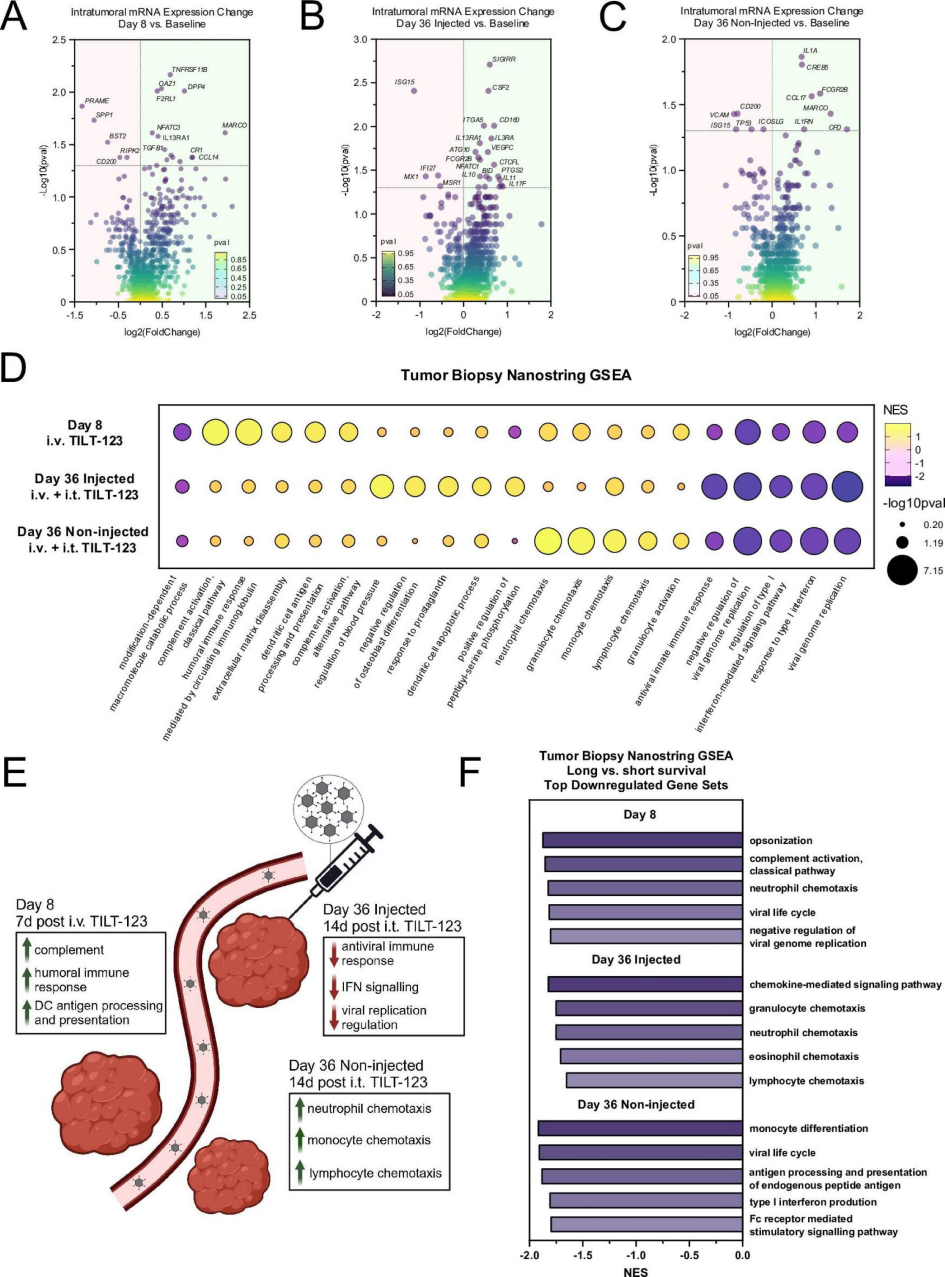
(A) Tumor transcriptional changes from baseline to day 8. (**B**) Tumor transcriptional changes from baseline to day 36 injected tumors. (**C**) Tumor transcriptional changes from baseline to day 36 non-injected tumors. (**D**) Gene set enrichment analysis of transcriptional changes from baseline to day 8, day 36 injected tumors and day 36 non-injected tumors. Net Enrichment Score (NES) and p value shown. (E) Graphical representation of key transcriptomic tumor findings. (**F**) Gene set enrichment comparing tumor transcriptomics of patients with long overall survival vs short overall survival (cut-off for long/short survival 120 days, N=6 patients per group on day 8, N=5 patients per group on day 36), on days 8, 36 injected and 36 non-injected respectively. For comparisons shown in A–C, samples compared with paired tests, and for comparisons shown in F samples compared with unpaired tests.

Next, we aimed to study changes in mRNA levels after TILT-123 therapy. On day 8 (7 days after intravenous TILT-123), a strong upregulation of inflammatory transcripts (*MARCO, DPP4, TNFRSF11B, CR1*) was still seen, leading to enrichment in gene sets including complement activation, humoral immune response, and antigen processing (figure 3A,D). Similarly, top downregulation of transcripts included *PRAME, SPP1, BST2*, leading to downregulation of gene sets related to negative regulation of virus replication and type I interferon response (figure 3A,D). These results suggest that intravenous TILT-123 delivery can induce proinflammatory changes in the tumors, with concurrent inhibition of antiviral responses, possibly arising from already cleared adenoviral infection or known adenoviral mechanisms promoting immune escape, such as the early adenoviral protein E1A.^26^ Regarding injected tumors on day 36, surprisingly few proinflammatory transcripts were upregulated (figure 3B). Top upregulated transcripts in injected tumors on day 36 included *SIGIRR*, *CSF2*, *CD160* and *IL3RA*, leading to upregulation of gene sets related to blood pressure regulation, osteoblast differentiation and prostaglandin response after enrichment (figure 3D). However, top downregulated gene sets in day 36 injected tumors included sets related to virus replication and antiviral responses, reminiscent of a completed immune response (figure 3D).

We next analyzed the transcripts of non-injected tumors from day 36. Surprisingly, top transcripts of non-injected tumors of day 36 included proinflammatory transcripts *IL1A*, *CREB5*, *FCGR2B* and *CCL17* (figure 3C), similar to top transcripts of day 8. After gene set enrichment, top upregulated sets associated with early immune response, such as neutrophil migration, granulocyte chemotaxis and lymphocyte chemotaxis (figure 3D). Conversely, downregulated gene sets included sets similar to day 8, including type-I interferon signaling viral genome replication (figure 3D), possibly suggesting that viral infection and early immune response were now occurring in the non-injected tumors, perhaps due to viral dissemination to non-injected metastases. A summarizing graphic showing the most relevant findings is shown in figure 3E.

To assess if patients benefitting from the therapy exhibited different tumor transcriptional changes, we compared patients with long overall survival to short overall survival. Patients were considered having short survival if they were alive less than 4 months (120 days) after enrolment, since the trial enrolled patients with an expected life expectancy of 3 months or more. Remarkably, at each time point of tumor sampling, patients with long overall survival showed upregulation of gene sets not related to inflammatory changes, such as gastrulation, somite development and digestive tract development (online supplemental figure 3). Conversely, when looking at downregulated gene sets, patients who showed longer overall survival after therapy showed downregulation of immune cell activity and chemotaxis (figure 3F), possibly suggestive of already completed immune response.

### TILT-123 mRNA transcripts in non-injected tumors correlate with longer overall survival and larger lymphocyte count decrease

**Figure 4:**
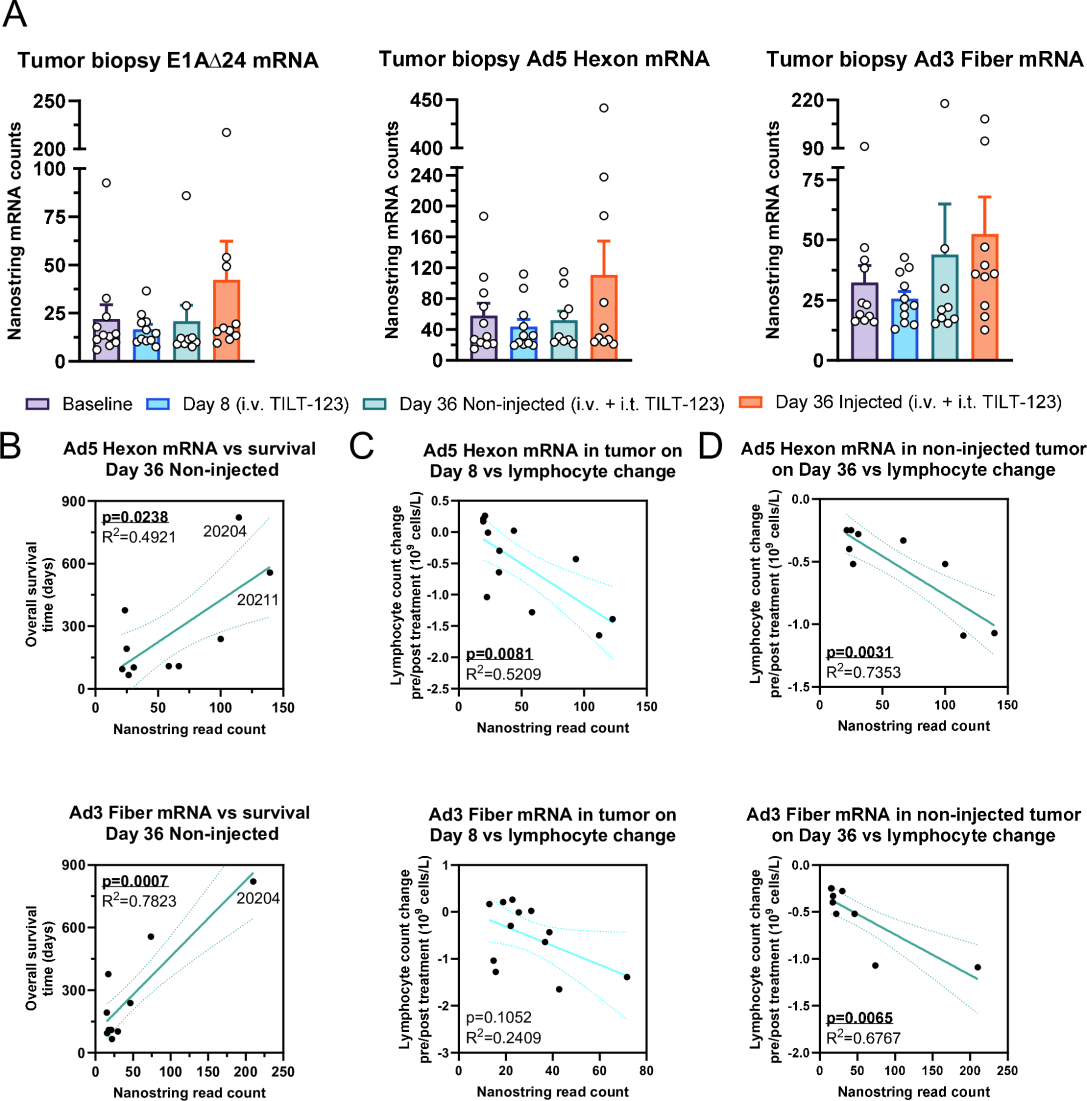
(A) Amounts of TILT-123 mRNA transcripts E1A∆24, hexon and fiber at baseline and in day 8, day 36 non-injected and day 36 injected tumors. Mean and SEM shown. (**B**) Correlation of TILT-123 mRNA transcripts in non-injected tumors to overall survival time. (**C**) Comparison of TILT-123 mRNA transcripts in day 8 samples to lymphocyte count decrease following therapy on day 8. (**D**) Comparison of TILT-123 mRNA transcripts in day 36 non-injected samples to lymphocyte count decrease following therapy on day 36. For (B–D) R^2^ for goodness of fit and p value for slope deviation from zero shown.

Next, we aimed to detect TILT-123 mRNA molecules in collected biopsies by including custom probes to the mRNA panel, which detected parts of the TILT-123 genome, specifically the transcripts of capsid constituents hexon and fiber, and the modified E1A with 24 bp deletion. Regarding these mRNA probes, no significant upregulation could be seen, although injected tumors on day 36 showed largest number of viral transcripts (figure 4A). When correlating the transcript level to overall survival, we observed a significant correlation of hexon and fiber transcripts with overall survival in day 36 non-injected lesions (figure 4B). Exceptionally, high mRNA transcripts of hexon and fiber were seen for patients 20204 and 20211 surviving 821 and 557 days, respectively. No similar correlation could be seen for day 8 or day 36 injected transcript counts (online supplemental figure 4A,B), suggesting that active virus presence in non-injected tumors was key for beneficial treatment outcomes, possibly due to successful intravenous spread between metastases.

Next, we aimed to assess if viral mRNA counts explained the cyclical peripheral lymphocyte count changes. Interestingly, when analyzing samples collected from day 8 and from day 36 non-injected lesions, higher numbers of virus transcripts pretreatment correlated with larger lymphocyte count decrease post-treatment (figure 4C,D). Interestingly, no similar correlation could be seen for day 36 injected lesions, possibly due to already cleared VPs (online supplemental figure 4C).

### TILT-123 induces immunological changes measurable from blood and serum

**Figure 5:**
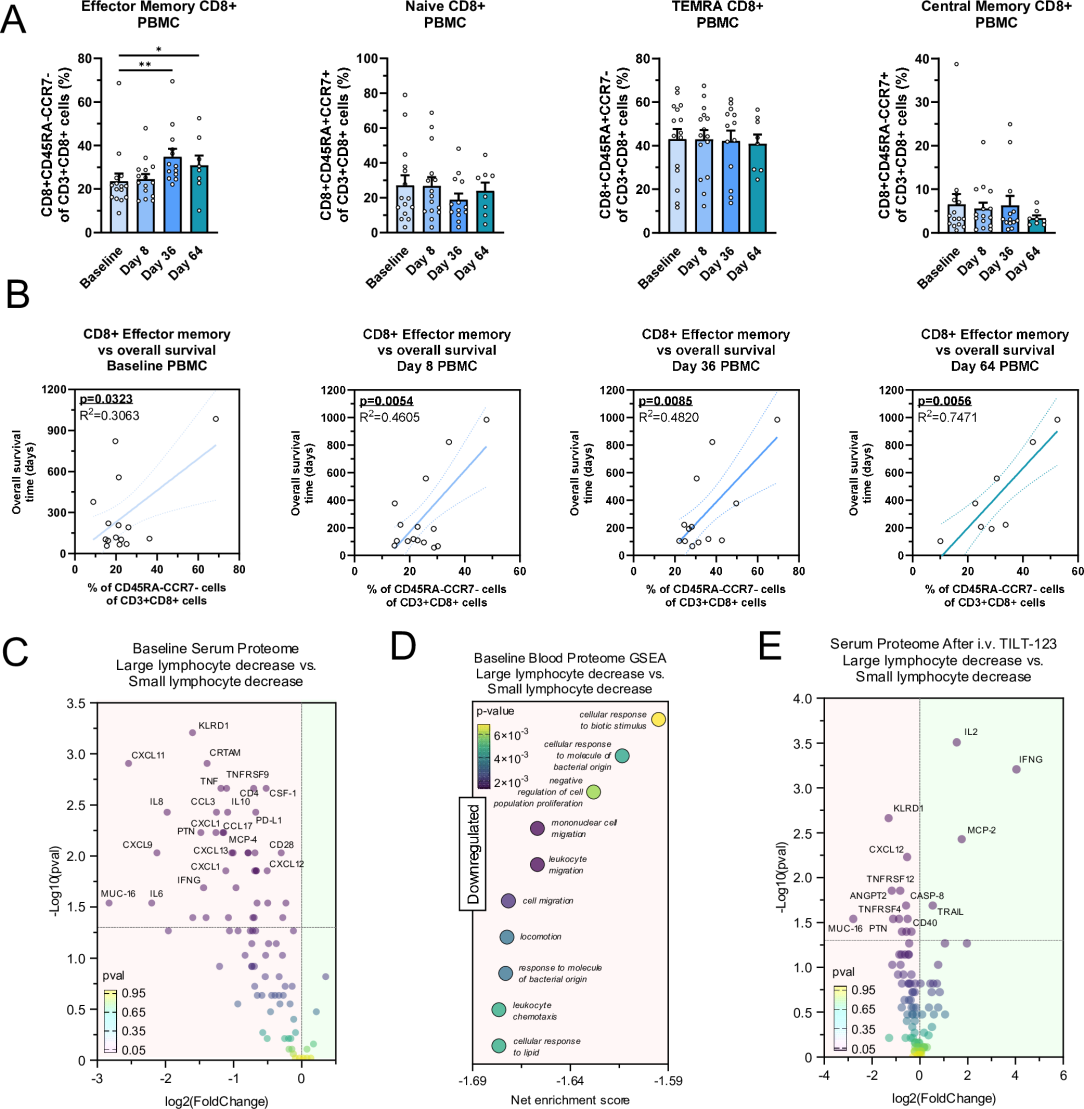
(A) Amount of effector memory, naïve, TEMRA and central memory CD8+T cells out of CD8+cells in PBMC samples across trial. Mean and SEM shown, groups compared with unpaired t-test. (**B**) Correlation of CD8+effector memory cell amount to overall survival. R^2^ for goodness of fit and p value for slope deviation from zero shown. (**C**) Differences in baseline serum proteomics in patients with large average lymphocyte decrease versus patients with small average lymphocyte decrease. (**D**) Downregulated serum proteomic pathways in patients with large average lymphocyte decrease versus patients with small average lymphocyte decrease. (**E**) Differences in serum proteomics 16 hours post intravenous TILT-123, comparing patients with large average lymphocyte decrease versus patients with small average lymphocyte decrease. For (C–E) patients allocated to small or large lymphocyte decrease by the median of all patients. For (C–E) groups compared with unpaired Wilcoxon rank-sum test. *p<0.05, **p<0.01. PBMC, peripheral blood mononuclear cells.

Next, we aimed to see if the observed intratumoral changes could also be seen in peripheral blood, in order to develop more easily translatable biomarkers. Thus, as immune reactivity after TILT-123 treatment seemed important for therapy success, we performed flow cytometry assessing memory subsets in PBMC samples (online supplemental figure 5A). TILT-123 therapy induced a significant increase in the CD8+effector memory subset at days 36 and 64 (p=0.0014 and p=0.0357, figure 5A). No similar changes could be seen for other CD8+memory subsets (figure 5A) or CD4+memory subsets (online supplemental figure 5B). When correlating CD8+effector memory cell amounts to overall survival, higher amounts of effector memory CD8+T cells correlated with longer overall survival at all time points (figure 5B), with better correlation at later time points, suggesting a benefit from a memory response to overall survival.

Further, we aimed to assess if we could see differences in baseline proteomics in patients who developed a large lymphocyte count decrease. Patients were split into large decrease or small decrease by the median percentage decrease. Interestingly, before therapy, patients who later developed a larger lymphocyte decrease showed a significantly downregulated serum proteome (figure 5C). Gene set analysis showed significant downregulation of immune response gene sets related to bacterial origin, mononuclear cell migration and leukocyte migration (figure 5D). However, 16 hours after intravenous TILT-123, patients who developed a larger lymphocyte decrease showed markedly more interferon-gamma (IFNg) and IL-2 in the serum, suggestive of a potent immune response (figure 5E).

### Patients with decrease in peripheral lymphocyte count post-TILT-123 injection present longer overall survival

**Figure 6:**
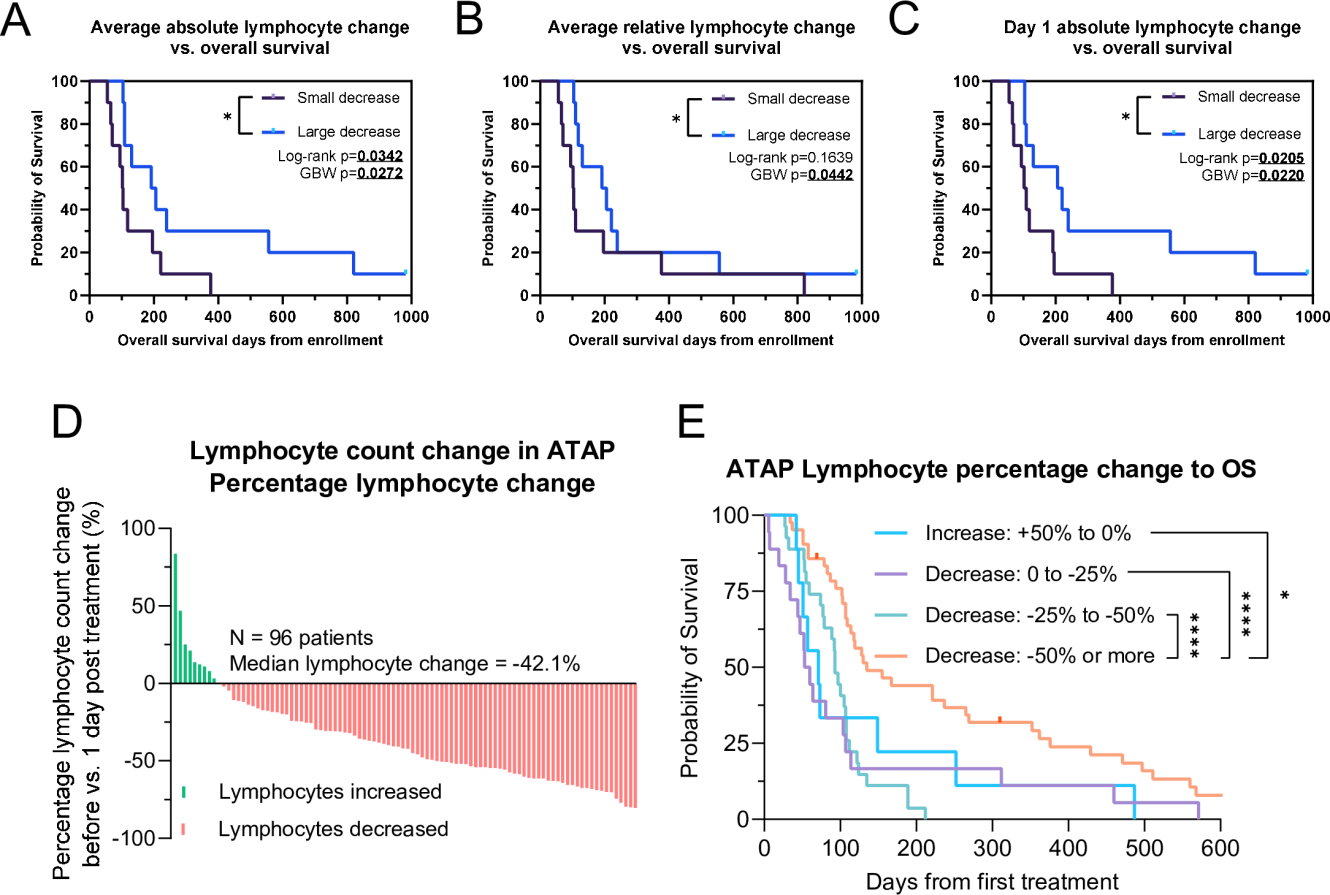
(A) Kaplan-Meier survival analysis of patients with large versus small average absolute lymphocyte decrease. (**B**) Kaplan-Meier survival analysis of patients with large versus small average relative lymphocyte decrease. (**C**) Kaplan-Meier survival analysis of patients with large versus small average absolute lymphocyte decrease on day 1. (**D**) Lymphocyte count change between pretreatment and post-treatment in validation dataset of ATAP patients. n=96 patients. (**E**) Kaplan-Meier survival analysis of overall survival (OS) in different levels of lymphocyte count change in confirmatory dataset. For graphs (A–C) patients split by median lymphocyte decrease, n=10 patients per group. For (A–C) groups compared with logrank test and Gehan-Breslow-Wilcoxon (GBW) test. For (E) groups compared with logrank test. *p<0.05, ****p<0.0001.

Finally, we assessed if lymphocyte count decrease could predict overall survival. For this analysis, patients were split the median change in lymphocyte count, thus allocating 10 patients to each group. Average absolute lymphocyte count decrease (10^9^ cells/L) predicted overall survival (p=0.0272, figure 6A), as did relative (%) change (p=0.0442, figure 6B). Of note, absolute lymphocyte count decrease already at day 1 was able to predict overall survival (p=0.0220, figure 6C), suggesting that peripheral blood lymphocyte count decrease is able to capture patients benefitting from the therapy already at an early time point.

### Confirmatory analysis in an external validation set

To validate our findings, we reanalyzed patients treated in the ATAP between 2007 and 2012, where 290 patients received different oncolytic adenoviruses in a personalized treatment setting. More in-depth studies on ATAP patient characteristics, treatments and outcomes have been published previously.^27^,^30^ 96 patients were available with pretreatment lymphocyte counts matched to lymphocyte count 1 day after first treatment. Like all patients in ATAP, patients were of advanced disease and refractory to existing standard oncological treatment, representing a similar patient demographic as TUNIMO. Summarized patient demographics are shown in online supplemental table 5. As in TUNIMO, most patients in ATAP developed a lymphocyte decrease 1 day after therapy, with a median decrease of 42.1%. A graphical presentation of lymphocyte count change in ATAP 1 day after therapy is shown in figure 6D. When comparing lymphocyte count change and overall survival time, patients with largest lymphocyte count change showed longest overall survival (figure 6E), confirming our findings of lymphocyte count decrease as a biomarker for oncolytic adenovirus treatment success.

## Discussion

Development of different immunotherapeutics has led to trials assessing efficacy in multiple cancer types. Although responses are seen regularly in melanoma and other cancer types, it is also clear that immunotherapy does not benefit all patients equally. Thus, identifying the right patient and tumor characteristics predictive of response, also known as biomarkers, is of key importance for the field. Biomarkers would allow selection of patients for immunotherapy who possess identified biomarkers while considering alternative therapies for patients without.

Engineered OV therapy has been tested in humans for about 25 years. However, in contrast to immune defective murine models, relatively little is still known about the mechanism of action of OVs in humans. There are major differences between mouse models and humans, and it might be that preclinical models are not able to capture the intricacies of the human immune system, often with over 60 years of exposure to various viruses prior to cancer onset. There are also other major differences between models and humans, including tumor microenvironment, physical size of animals and inherently different biology of rodents compared with humans. Thus, much discussion is still ongoing regarding fundamental aspects of OVs, from virus strain and genetic elements to administration route. Most likely, the ‘optimal’ OV does not exist, instead, the ‘optimal virus’ for each patient is dictated by the patient itself: a patient presenting with a long history of adenoviral conjunctivitis episodes most likely mounts a different type of immune response to adenovirus-based therapeutics in comparison to a patient with a history of oral herpes infections but no adenovirus infections. Thus, as a field it is critical to identify drivers behind responses in clinical trials, of which there are many ongoing currently in the OV space. Our results here aimed to identify markers of response in the TUNIMO trial, where patients with advanced solid tumors were treated with TILT-123, an oncolytic adenovirus encoding TNFa and IL-2. We also aimed to produce information regarding the mechanism of action of TILT-123 in humans.

We were able to show that lymphocyte decrease correlates with tumor size decrease and overall survival. In essence, a larger lymphocyte decrease predicted a better outcome for the patient. Lymphocyte decrease (or lymphopenia) is a common phenomenon associated with clinically relevant viral infections, and this transient fluctuation is often attributed to immune system activation and lymphocyte trafficking.^31 32^ Limited research relating to adenoviruses and lymphocyte count decrease exists, but some research has been conducted in different transplant settings, where adenoviral infections can be problematic. In these scenarios, acute adenovirus infections are often accompanied by lymphopenia, which often resolves after viral clearance.^33 34^ Additionally, multiple different OV trials have reported transient lymphopenia as an adverse event, but the relation to lymphocyte decrease (above or below the count defined as lymphopenia) to treatment response has not been thoroughly studied.^35^,^38^ It should be highlighted that lymphocyte decrease associated with OVs is generally non-symptomatic and self-resolving, and does not seem to predispose to microbial infection. This is an important distinction from chemotherapy-induced leukopenia or neutropenia, where bone marrow suppression can lead to opportunistic infections. We were able to show that lymphocyte count decrease 1 day after therapy predicted both favorable imaging results and longer overall survival in TUNIMO. Furthermore, we validated our findings in a set of 96 patients with advanced solid cancers treated with 10 different oncolytic adenoviruses, providing compelling evidence that the findings reported here are not restricted to TILT-123, but might be true for oncolytic adenoviruses in general.

We found that lymphocyte decrease in blood was correlated with increased amounts of T cells and NK cells in tumors when assessed with multiplexed immunofluorescence, and that increased intratumoral TILT-123 mRNA transcripts correlated with larger lymphocyte count decrease. Furthermore, we showed that lymphocyte count decrease is dictated by inherent patient characteristics at baseline and after therapy, where baseline serum of patients with large lymphocyte count decreases during therapy was markedly less immunologically active, but conversely post-therapy serum showed close to 16-fold larger interferon responses, suggestive of stronger antiviral response. In terms of real-life applicability, absolute lymphocyte counting is widely available in all hospitals, and easily included in standard safety laboratory testing. Our results suggest that lymphocyte count monitoring can be a cost-effective method to select patients for continued oncolytic adenovirus therapy.

Regarding mechanism of action of TILT-123 in humans, our results show that TILT-123 administration leads to lymphocyte accumulation in tumors after the intravenous dose, but the increase is not significant after intratumoral dosing. There could be at least three reasons for this effect: timing, route of administration and order of administration. First, the biopsies were collected pretreatment, thus the day 8 sample was collected 7 days after the intravenous administration, whereas the day 36 samples were collected 14 days after the day 22 intratumoral administration. This difference in timeline could be critical regarding the results since viral infections are often cleared quickly, and the day 36 sampling might have missed the window for detecting lymphocyte increase in tumors. Supportive of this hypothesis is the observation of a downregulated immune response in biopsy transcriptomics in day 36 injected tumors. Second, it is possible that intravenous dosing is more efficacious in transducing multiple tumors. The chimeric knob region and dual selectivity devices of TILT-123 have been designed to facilitate intravenous delivery, while intratumoral delivery has known challenges in the clinical setting, such as difficulty in injecting viable tumor regions and accessibility of deep lesions.^39 40^ Third, due to the trial protocol, intravenous injection always occurred prior to intratumoral injection, which might be relevant regarding the strength and status of the antiviral response.

Additionally, we also showed that higher expression of PD-L1 in stromal immune cells correlated with longer overall survival. Research in head and neck cancer patients has shown that PD-L1 expression on immune cells is a better prognostic factor than PD-L1 expression on cancer cells.^41^ Little is known of the stromal interactions of OVs in humans, but since most human cancers are stroma-rich, research on virus–stroma interactions warrants further investigation.^42^

Like all research, our findings have caveats. Findings regarding the mechanism of action of TILT-123 were from 20 patients with various types of advanced solid tumors, constituting a heterogenous group of patients. Nevertheless, this can also be seen as a strength, as this population is perhaps reflective of a ‘real-world situation’, and if a finding is present in a mixed population, it is more likely to be an important general phenomenon instead of restricted to a specific situation. However, especially the analyses relating to overall survival are in risk of bias due to inherent differences in aggressiveness of different solid tumors included in this phase I trial. Another caveat is that not all sample types were available from all patients and all time points. However, we tried to avoid any bias by including multiple parallel assays, to validate findings of one assay with the other. Regardless, 20 patients are still a limited dataset, and findings presented here should be repeated with a larger and possibly more homogenous patient population. As the first step toward independent validation, we studied 96 patients treated in a program not related to TUNIMO and saw the same association between lymphocyte decrease and survival. However, many other assays, such as IHC and transcriptomics were not able to be validated with the archival data, and future studies should validate our findings in other trials of oncolytic adenoviruses, especially in analyses where outliers were seen, such as viral mRNA analysis.

In summary, we have identified multiple factors which predict imaging outcomes and/or long survival in advanced solid tumor patients treated with TILT-123. Future research will indicate if these mechanisms of action apply to also other types of OVs. The key finding of the study is that lymphocyte decrease predicts imaging outcome and survival. This test is quick, simple and can be performed in any hospital or clinic, making it a potentially valuable practical biomarker.

## supplementary material

10.1136/jitc-2024-010493 online supplemental file 1

## Data Availability

Data are available on reasonable request.
