# Supplementary material for: Transient lymphocyte count decrease correlates with oncolytic adenovirus efficacy in humans: mechanistic and biomarker findings from TUNIMO phase I trial
Source: J Immunother Cancer. 2025 Jan 27;13(1):e010493. doi: 10.1136/jitc-2024-010493 (PMC11772932; doi:10.1136/jitc-2024-010493)
Supplement: online supplemental file 1 [file jitc-13-1-s001.pdf]

PET CRITERIA USED IN TUNIMO (NCT04695327)	
Complete metabolic response – CMR	Complete resolution of FDG activity within measurable lesions and all reliably assessable lesions to background levels. No new FDG-avid lesions in pattern typical of cancer*. Lymph nodes may remain metabolically active due to immune response (activated lymphocytes take up FDG).
Partial metabolic response – PMR	> 30 percent decrease in FDG activity measured as the summed SUVmax of measurable lesions (up to five lesions, max 2/organ). No new FDG-avid lesions in pattern typical of cancer*.
Minor metabolic response – MMR	10-29 percent decrease in FDG summed SUVmax. No new FDG-avid lesions in pattern typical of cancer*.
Stable metabolic disease – SMD	0-9 percent decrease or up to < 30 percent increase in FDG summed SUVmax. No new FDG-avid lesions in pattern typical of cancer*.
Progressive metabolic disease – PMD	≥ 30 percent increase in FDG summed SUVmax in pattern typical of tumor, or new clearly FDG-avid clinically significant lesions in pattern typical of cancer*. Increase in metabolic activity in lymph nodes should not result in PMD if no progression is detected elsewhere, since it might reflect immunological activation and not progression.
* "clinically significant lesions in pattern typical of cancer" defined as lesions associated with a CT abnormality most consistent with cancer (≥ 2 cm in diameter), and clearly not because of inflammation of infection or related to treatment response.	

Supplementary Table 1. PET-criteria used in TUNIMO.

	Panel A	Panel B	Panel C
**First round staining**			
**TSA-488**	**R-anti-CD56**	**R-anti-CD4**	**M-anti-CD68**
Antibody clone	CM;156R-94	Abcam ab133616	CellMarque 168M-94
**TSA-555**	**M-anti-CD8**	**M-anti-CD20**	**R-anti-CD11c**
Antibody clone	Dako M7103	Thermo MS-340	Abcam ab52632
**Alexa-647**	**M-anti-PD1**	**M-anti-FoxP3**	**R-anti-PD-L1**
Antibody clone	LSBio LSB12784	Abcam ab20034	CST 13684
**Alexa-750**	**R-anti-GranzymeB**	**R-anti-GranzymeB**	**M-a-CD45**
Antibody clone	Abcam ab4059	Abcam ab4059	Dako M0701
**Second round staining**			
**Alexa-647**	**M-a-CD45**	**M-a-CD45**	**R-anti-CD16**
	Dako M0701	Dako M0701	CM 116R-14
**Alexa-750**	**R-anti-panEpi cocktail**	**R-anti-panEpi cocktail**	**M-anti-panEpi cocktail**
	R-a-Ecadherin; CST 3195	R-a-Ecadherin; CST 3195	M-anti-panCK; Abcam; ab7753
	R-a-pan Cytokeratin; Abcam ab9377	R-a-pan Cytokeratin; Abcam ab9377	M-anti-panCK; Invitrogen; MA5-13156
			M-anti-E-Cadherin; BD; 610182

Supplementary Table 2. Immunohistochemistry staining panels, antibodies, clones and vendors used.

Target	Clone	Conjugation	Dilution	Vendor	Catalogue number
CD3	UCHT1	BV711	1:100	BD Biosciences	563725
CD4	RPA-T4	BV605	1:100	BD Biosciences	562658
CD8	SK1	BV510	1:100	BD Biosciences	563919
CD45RA	HI100	AF700	1:100	BD Biosciences	560673
CD197 (CCR7)	G043H7	PE-Cy7	1:100	Biolegend	353226
DNA (Dead cell discrimination)		7-AAD	1:100	Biolegend	420404

Supplementary Table 3. Flow cytometry staining panel, antibody clones, dilutions, vendors and catalogue numbers used.

Gene	Position	Target sequence	NSID
Hadv5_hex	333-432	ATGCAATTTTTCTCAACTACTGAGGCAGCCGCAGTCAATGGTGATAACTTGACTCCAAAAGTGGTATTGTATAGCGAAGACGTGGATATAGAAACTCCAG	FN813606.1:332
Hadv3_fiber_knob	362-461	AATGCTACTACAAAGCAAGCGATGGTGCCCTTTTTCCGTTGGAAGTTACTGTTATGCTTAATAAACGCCTGCCAGATAGTCGCACATCCTATGTTATGAC	KF279606.1:361
Hadv5_E1A/B_mod	295-394	CCCGAGCAGCCGGAGCAGAGAGCCTTGGGTCCGGTTTCTATGCCAAACCTTGTACCGGAGGTGATCGATCCACCCAGTGACGACGAGGATGAAGAGGGTG	Hadv5_e1a_mod.1:294

Supplementary Table 4. Custom mRNA probes used in mRNA panel.

	NUMBER (PERCENTAGE OF ALL) OR MEDIAN
**TUMOR TYPE**	
OVARIAN CANCER	14 (14.6%)
COLORECTAL CANCER	13 (13.5%)
LUNG CANCER	10 (10.4%)
PANCREATIC CANCER	9 (9.4%)
SARCOMA	9 (9.4%)
BREAST CANCER	8 (8.3%)
HEAD AND NECK CANCER	5 (5.2%)
MELANOMA	5 (5.2%)
PROSTATE CANCER	5 (5.2%)
MESOTHELIOMA	4 (4.2%)
NEUROENDOCRINE CANCER	3 (3.1%)
BILE DUCT CANCER	2 (2.1%)
ENDOMETRIAL CANCER	2 (2.1%)
STOMACH CANCER	2 (2.1%)
ESOPHAGEAL CANCER	1 (1.0%)
KIDNEY CANCER	1 (1.0%)
LIVER CANCER	1 (1.0%)
SMALL INTESTINE CANCER	1 (1.0%)
UROTHELIAL CANCER	1 (1.0%)
**AGE, MEDIAN**	60
**SEX**	
FEMALE	49 (51.0%)
MALE	47 (49.0%)
**WHO**	
0	8 (8.3%)
1	40 (41.7%)
2	35 (36.5%)
3	13 (13.5%)

Supplementary Table 5. Demographics of patients included in the external validation set.

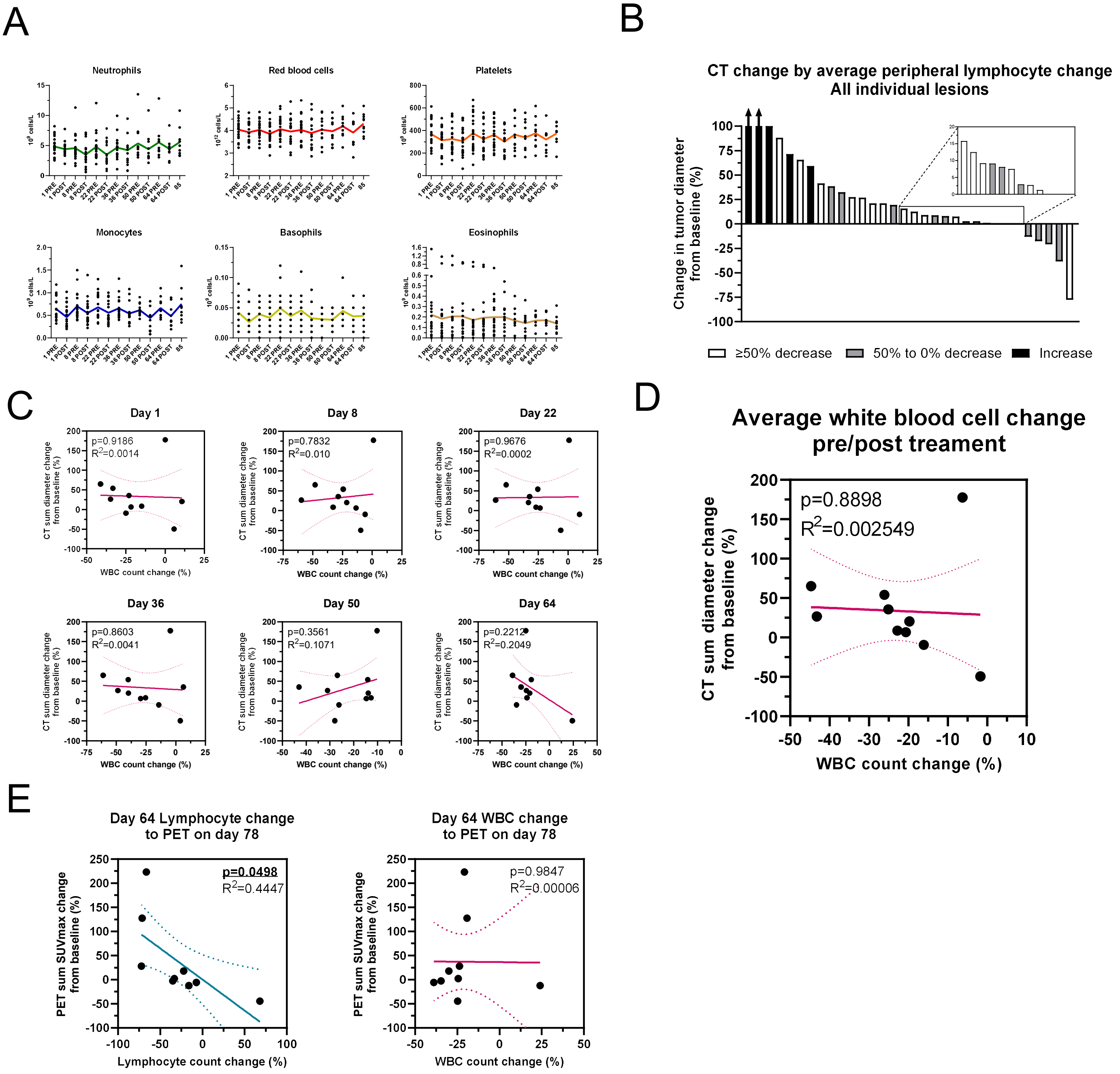

Supplementary Figure 1. A. Changes in neutrophils, red blood cells, platelets, monocytes, basophils, and eosinophils across trial. All individual datapoints shown, means presented with colored line. **B.** Waterfall plot of all CT imaged lesions classified by average peripheral lymphocyte count change. **C.** Correlation of WBC count change on each treatment day to CT tumor diameter change. **D.** Correlation of average WBC count change to CT tumor diameter change. **E.** Day 64 lymphocyte and WBC count change to PET sum SUVmax change at day 78. For C-E, R2 for goodness of fit and p-value for slope deviation from zero shown.

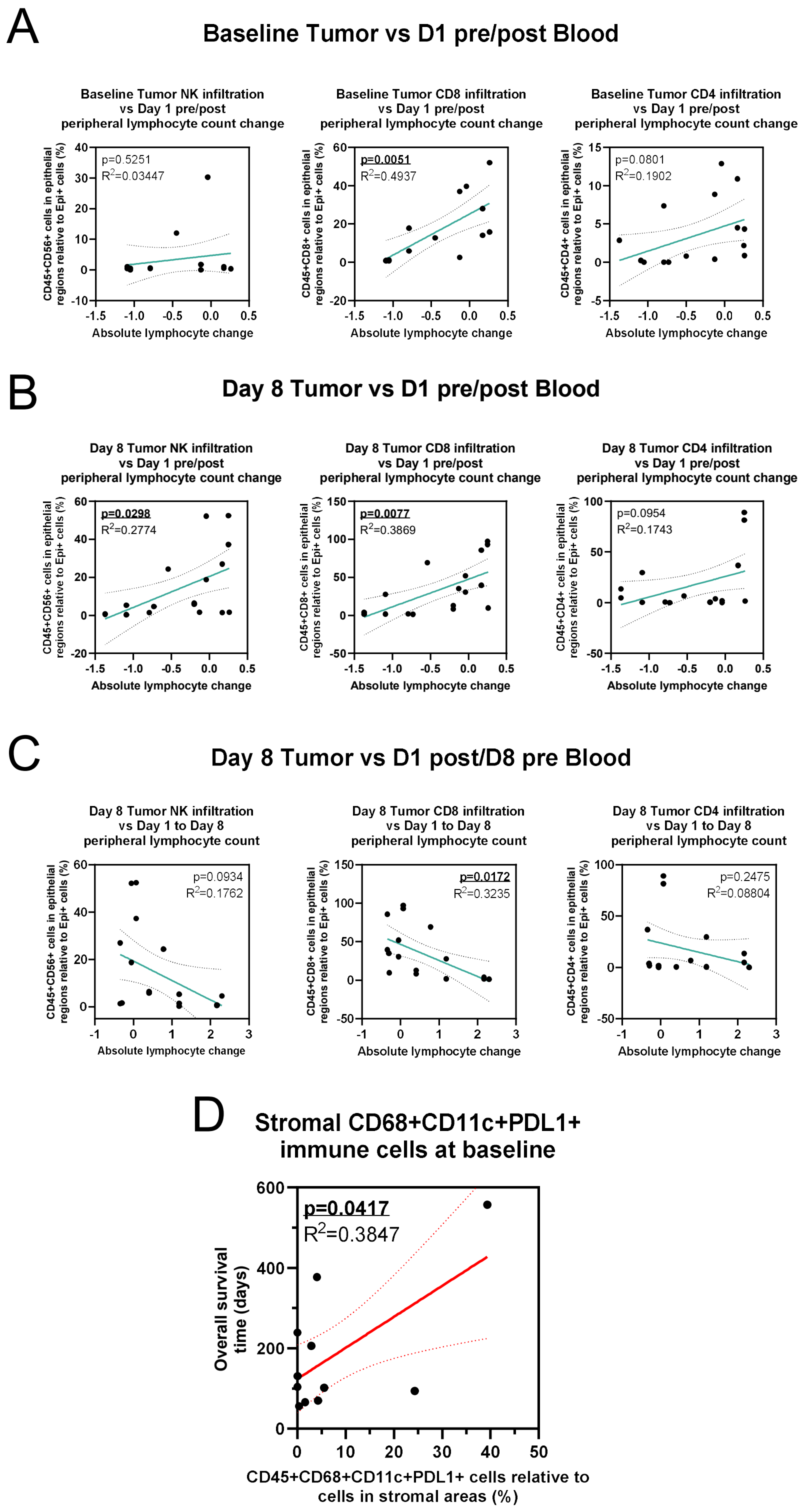

Supplementary Figure 2. A. Baseline tumor NK cell, CD8+ T cell and CD4+ T cell infiltration vs blood lymphocyte count change after day 1 TILT-123 administration. **B.** Day 8 tumor NK cell, CD8+ T cell and CD4+ T cell infiltration vs blood lymphocyte count change after day 1 TILT-123 administration. **C.** Day 8 tumor NK cell, CD8+ T cell and CD4+ T cell infiltration vs blood lymphocyte count change from day 1 post-treatment to day 8 pre-treatment. **D.** Stromal infiltration of CD45+CD68+CD11c+PDL1+ cells vs overall survival. For all graphs, R^2 for goodness of fit and p-value for slope deviation from zero shown.

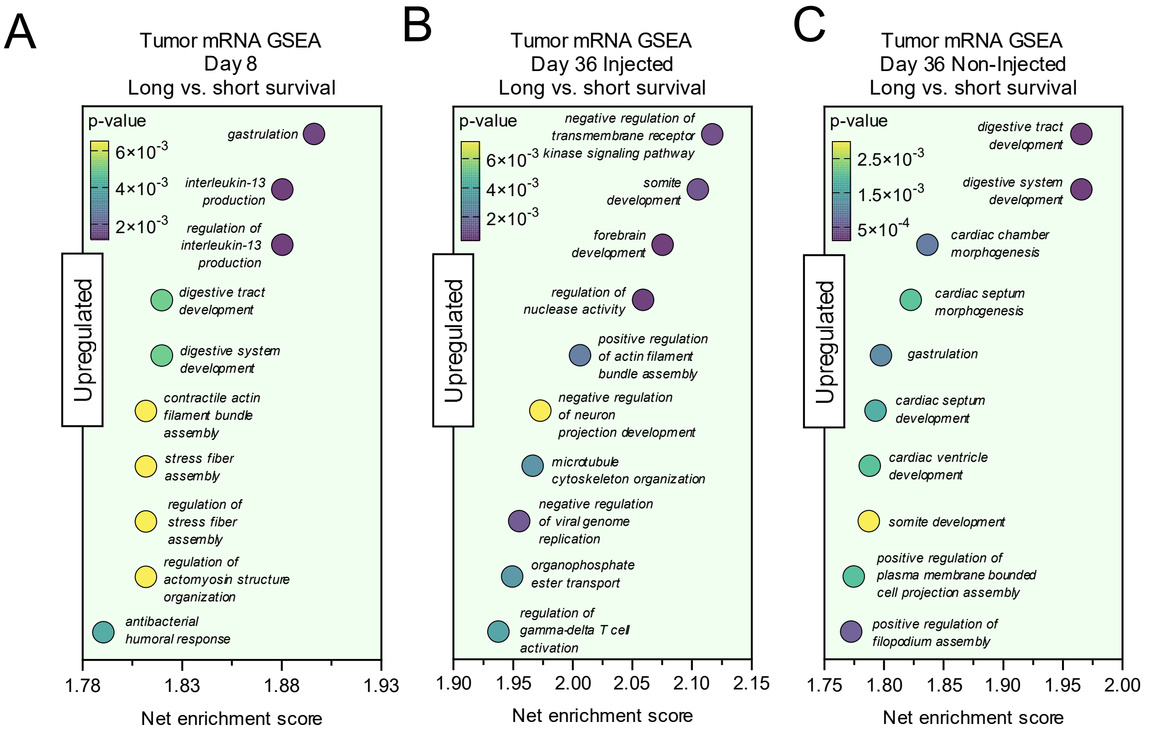

Supplementary Figure 3. Gene set enrichment analysis of tumor transcriptional changes comparing patients with long overall survival to short overall survival, upregulated gene sets in **A.** Day 8 tumor samples, **B.** Day 36 injected samples and **C.** Day 36 non-injected tumor samples. Gene set enrichment analysis from non-paired comparison of transcript counts.

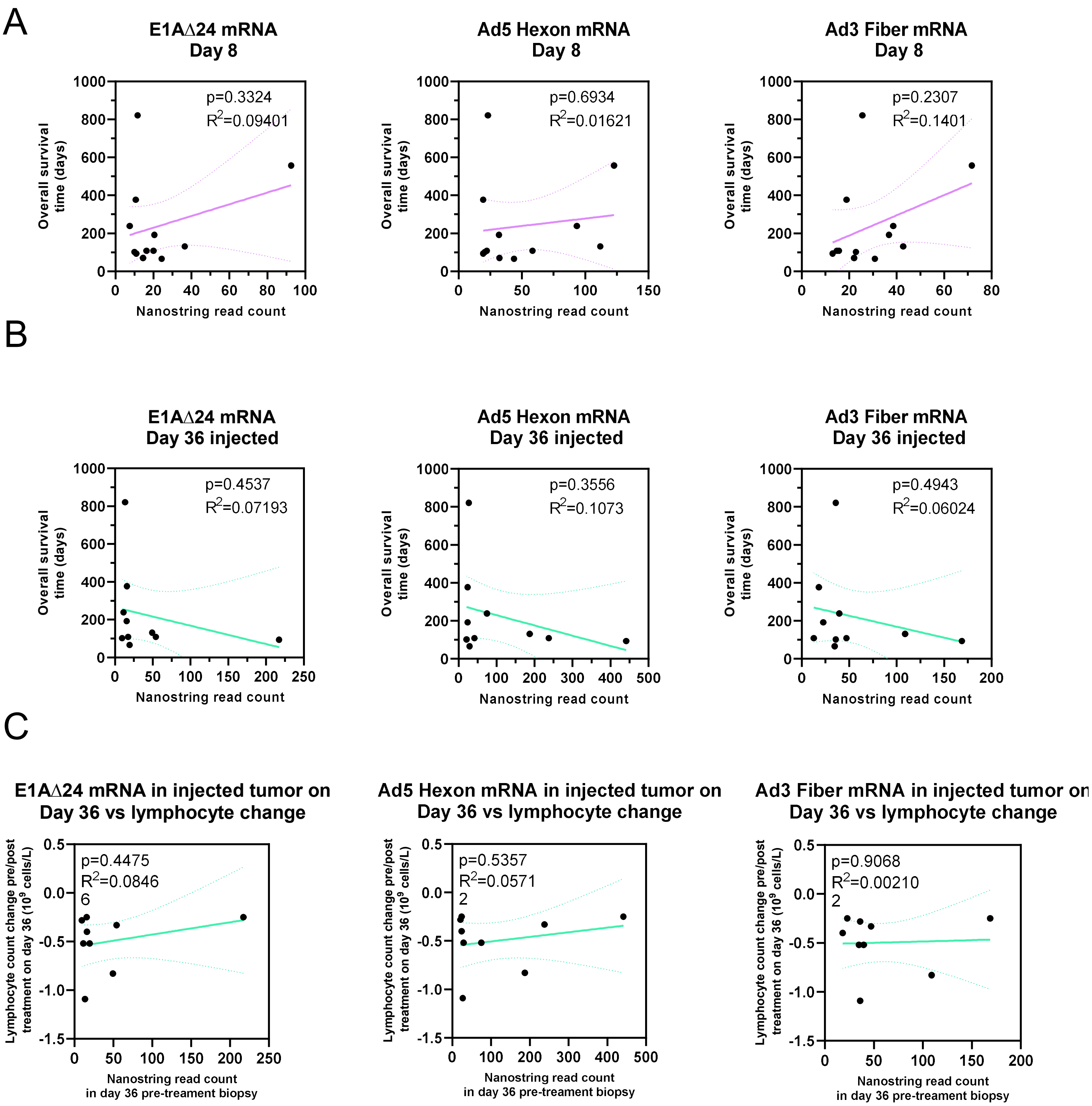

Supplementary Figure 4. A. Comparison of intratumoral TILT-123 mRNA transcripts in day 8 samples to overall survival. **B.** Comparison of intratumoral TILT-123 mRNA transcripts in day 36 injected samples to overall survival. **C.** Comparison of intratumoral TILT-123 mRNA transcripts in day 36 injected samples to lymphocyte count change following therapy on day 36. For all graphs, R^2 for goodness of fit and p-value for slope deviation from zero shown.

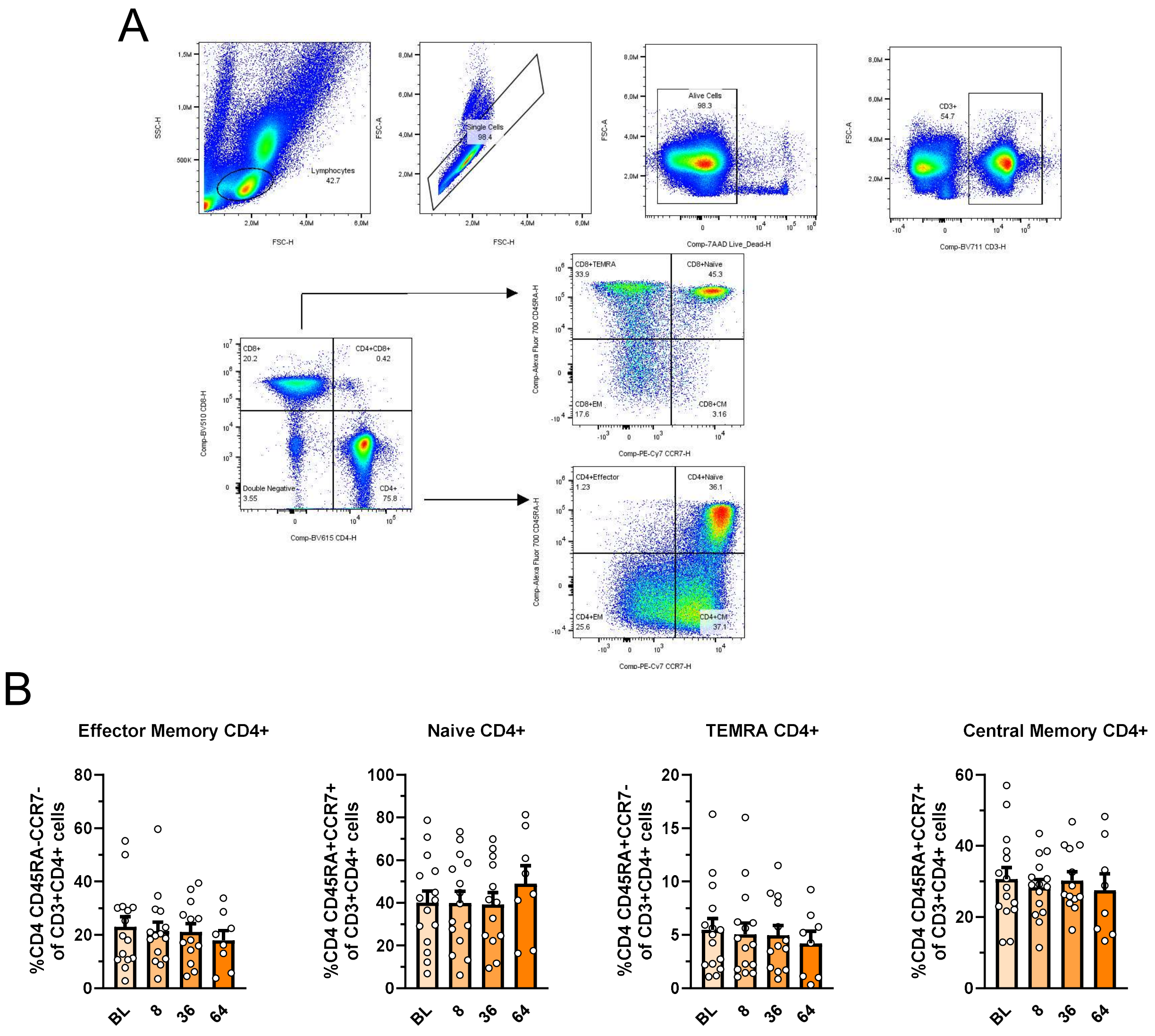

Supplementary Figure 5. A. Flow cytometric gating scheme for PBMC memory subsets. **B.** Amounts of CD4+ effector memory, naïve, TEMRA and central memory cells in PBMCs across trial. Mean and SEM shown. Groups compared with unpaired t-test.
